# *Burkholderia cepacia* infection in a non-cystic fibrosis patient: an arcane presentation

**DOI:** 10.1099/acmi.0.000222

**Published:** 2021-04-15

**Authors:** Sufyan Ibrahim, Haritha Madigubba, Himanshu Y. N, Kiran Chawla

**Affiliations:** ^1^​ Kasturba Medical College, Manipal, Manipal Academy of Higher Education, Manipal, Karnataka, India; ^2^​ Department of Microbiology, Kasturba Medical College, Manipal, Manipal Academy of Higher Education, Manipal, Karnataka, India

**Keywords:** acute exacerbation, antibiotic resistance, *Burkholderia cepacia*, ceftazidime, COPD, cystic fibrosis

## Abstract

**Introduction:**

*
Burkholderia cepacia* is an aerobic, Gram-negative bacillus, which exhibits innate resistance to multiple antibiotics and disinfectants. Although it is a chronic colonizer of the respiratory tract, it may rarely present with fatal necrotizing pneumonia-like features in immunosuppressed individuals, as those with chronic granulomatous disease, or patients with significant pulmonary compromise, like cystic fibrosis.

**Case Presentation:**

A 76-year-old male presented with complaints of breathlessness, cough with mucoid expectoration and fever for 3 days. He had a history of coronary artery disease, chronic obstructive pulmonary disease (COPD), diabetes mellitus and hypertension, under treatment. Pulmonary function tests were suggestive of very severe obstruction (FEV1/FVC was 55 %). So, clinical diagnosis of acute exacerbation of COPD was established. Sputum culture grew *B. cepacia *. The patient was treated with ceftazidime and meropenem along with inhalational bronchodilators and steroids, and showed symptomatic response to therapy.

**Conclusion:**

There is paucity of the literature describing *B. cepacia* as a potential cause for acute exacerbations in relatively common clinical conditions, such as COPD. This case report highlights the speculation of this rare possibility, thereby alerting a clinician dealing with such cases.

## Introduction

*Burkholderia cepacia* complex, previously called *Pseudomonas cepacia *, are a group of closely related oxidase positive, catalase producing, non-lactose fermenting, aerobic, motile, Gram-negative bacilli, infrequently appearing in case reports as causes of human infections ever since their discovery by Burkholder in the root of onion bulbs in 1950 [1, 2]. Owing to its innate resistance to many antibiotics and disinfectants, including polymyxin B, povidone iodine and chlorhexidine, the bacteria is frequently isolated as a nosocomial pathogen, causing fatal necrotizing pneumonia-like features, more so in patients with an underlying lung disease or those with immunodeficiency [3]. While clinicians would be vigilant enough to diagnose the said infection in cystic fibrosis patients considering its relatively high incidence in the patient subgroup, suspecting the pathogen as a cause of acute exacerbation of common illnesses like chronic obstructive pulmonary disease (COPD) or bronchiectasis needs pre-emption. The lack of sufficient cases in the literature for the same makes it all the more difficult to speculate such a rare aetiology for typical infectious exacerbations [4]. We herein present one such rare case of *B. cepacia* in a patient with COPD who was hospitalized due to type II respiratory failure.

## Case report

**Fig. 1.**
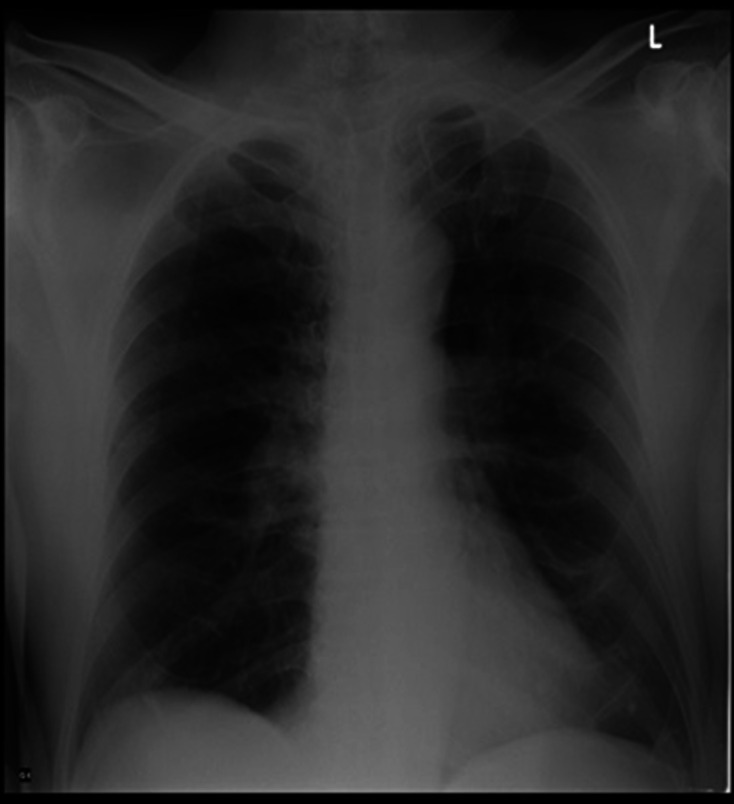
CXR showing hyperinflated lungs with patchy opacities in the right lung and small cavities bilaterally.

**Fig. 2.**
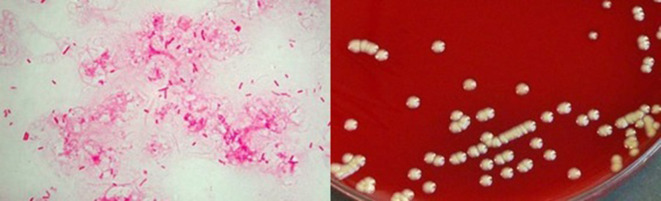
Gram stain (100X) showing Gram-negative bacilli. Growth on 5 % sheep blood agar showing rough and corrugated colonies.

**Fig. 3.**
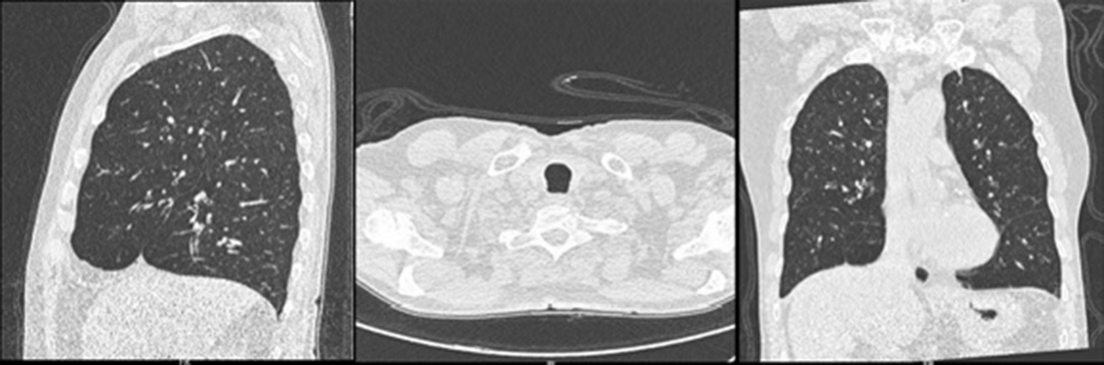
CT chest showed centriacinar emphysematous changes involving the bilateral lung parenchyma, predominantly the bilateral upper lobe parenchyma. Central tubular bronchiectasis with bronchial wall thickening is seen involving the segmental and lobar bronchi of the right middle and lower lobe, and lower lobe of the left lung parenchyma.

A 76-year-old male presented with complaints of breathlessness, cough with whitish mucoid, non-foul smelling and non-blood tinged expectoration, and fever for 3 days. He had a history of coronary artery disease, chronic obstructive pulmonary disease, diabetes mellitus and hypertension, under treatment. There was no history of loss of appetite, wheezing, haemoptysis, palpitations, chest pain, paroxysmal nocturnal dyspnoea, orthopnea or syncope. On physical examination, the patient was conscious and coherent. Pulse was 86 bpm, blood pressure was 130/80 mm of Hg and temperature was 101 °F. Respiratory examination revealed bilateral diffuse rhonchi with crepitations on auscultation. Investigations showed raised total leucocyte count (12 300 µl^−1^), neutrophils (75.8 %), lymphocytes (14.6 %) and ESR (49 mm at the end of first hour). SpO_2_ was 94 % on room air. Chest x-ray revealed hyperinflated lungs, consistent with emphysema, and with patchy reticular opacities in the middle zone of right lung (Fig. 1). Pulmonary Function Tests were suggestive of very severe obstruction (FEV1/FVC was 55 %). A clinical diagnosis of acute exacerbation of COPD was made. Gram staining of sputum revealed many pus cells with presence of Gram-negative rods, and sputum culture grew moist, creamish colonies on sheep blood agar (Fig. 2) that were later identified as *B. cepacia* based on battery of tests suggesting motile, oxidase positive, catalase positive bacilli. Decarboxylation of lysine was positive and demonstrated resistance to polymyxin B whereas arginine hydrolysis and ornithine decarboxylation were negative. The identification was also confirmed with MALDI-TOF. Staining for acid fast bacilli for two consecutive sputum samples was negative. Computerized tomography of chest confirmed centriacinar emphysematous changes with central, tubular bronchiectasis (Fig. 3).

### Outcome and follow up

Initially the patient was managed with inhalational and oral bronchodilators, and systemic steroids. But after the sputum culture grew *B. cepacia *, which was sensitive only to ceftazidime, aztreonam, cefepime and meropenem, the patient was started on ceftazidime and meropenem. A repeat chest X-ray performed 3 days after the patient was started on antibiotics showed clinico-radiological improvement. The injectable antibiotics were continued for 2 weeks. The patient was symptomatically better and was discharged in a stable condition with the advice to continue the oral antibiotics for 1 week, along with inhalational bronchodilators.

## Discussion

Human infections due to *B. cepacia* are rare, the affected patients usually morbid with chronic granulomatous illness, cystic fibrosis, drug addiction or immunocompromised states (cystic fibrosis being the most common), as cases with fulminating pneumonic infection along with fever and respiratory failure, and occasionally as septicaemia (known as ‘*cepacia* syndrome’) [3]. The gamut of clinical presentation may vary from superficial skin and soft-tissue infections to deep-seated infections like pneumonia, pyothorax, urinary tract infection, necrotizing fasciitis, but these are rare in immunocompetent individuals. *B. cepacia* has also frequently been encountered in nosocomial outbreaks due to contaminated disinfectants, nebulizer solutions, mouth wash, medical devices and intravenous solutions due to contamination of lipid emulsion stoppers – hence it becomes very important to diagnose it in order to prevent hospital-acquired infections and outbreaks in intensive care units [4]. Cases in hospitalized non-cystic fibrosis patients are rare, occurring mainly due to contaminated antiseptics, disinfectants, ventilators and other types of medical equipment with a documented evidence of person-to-person transmission [4, 5]. The organism being multi-drug resistant, cotrimoxazole is the drug of choice, used with or without ceftazidime or meropenem [6]. Patients with COPD have lesions, such as bronchiectasis, which may be niches for chronic colonization [4]. Use of organism-sensitivity directed, broad spectrum antibiotics with good penetration in the lung parenchyma may help control acute infectious exacerbations. [6]

We may hence speculate that *B. cepacia *, a chronic colonizer with innate resistance to multifarious classes of antibiotics, may cause infections in patients with lung disease other than cystic fibrosis, such as COPD, which was highlighted in our case. Hence, a clinician must be alerted to the possibility of infection by such a rare pathogen with multidrug resistance while dealing with acute exacerbations of common clinical condition like COPD.
